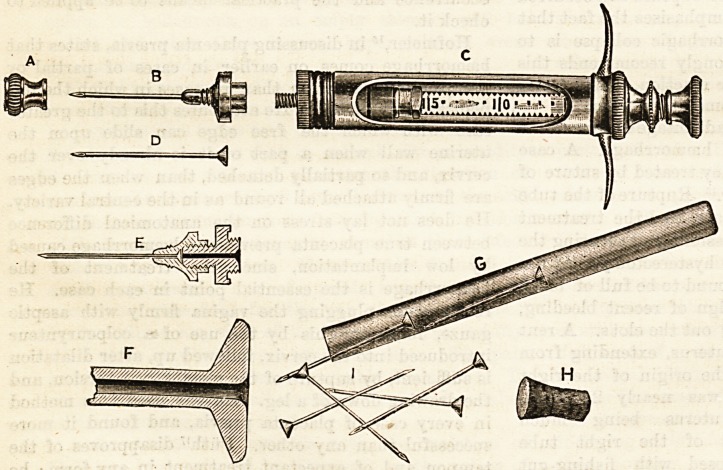# New Appliances and Things Medical

**Published:** 1897-11-06

**Authors:** 


### NEW APPLIANCES AND THINGS MEDICAL.

[We shall be glad to receive, at our Office, 28 & 29, Southampton Street, Strand, London, W.C., from the manufacturers, specimens of all new preparations and appliances which may be brought out from time to time.]

### ALUMINIUM HYPODERMIC CASE.

(Parke, Davis, and Co., 21, North Audley Street, Grosvenor Square, W., and Detroit, Mich.)

The new hypodermic case which we have before us is a modification of the improved hypodermic case by the same firm. Its distinguishing name in full is "Aluminium Hypodermic Case, No. 2." The merits of "The Improved Hypodermic Case" are now so well known that recapitulation is unnecessary; but in the new variety the needle and connections, which are designed to facilitate perfect asepsis, require some explanation. By means of the adapter (E) it is possible to make use of Schimmel's aseptic hypodermic needles, instead of fitting the needle on to the nozzle in the ordinary manner. The advantage of this is very obvious—that is to say, one and the same syringe can be used either for Schimmel's or for ordinary needles. By substituting Schimmel's needles for the ordinary kind, a leather washer is rendered unnecessary and a possible factor of contamination avoided. Beyond the question of cleanliness, there is the further advantage that the needle cannot become unsoldered, and a liberal supply can be carried without increasing the bulk. A vial of six needles can be purchased for 2s., and a separate needle employed for each patient without incurring any great outlay. The needles, being kept in a stoppered tube and previously sterilised at a temperature of 300 deg. F., are always ready for immediate use, free from rust and perfectly aseptic. The details of the syringe may be understood by studying the illustration which we append. A is the cap for adapter when syringe is not in use; B, loose hypodermic needle mounted in butt; D, Schimmel needle; E, longitudinal section of needle and adapter; F, magnified end of needle, showing central countersinking of the mounting to permit of easy introduction of wire after using; G, needles and tube; H, stopper for tube. There can be no doubt that this new syringe has immense advantages, and by the simple expedient of using a separate needle for each patient, which is hereby rendered possible and convenient, accidental inoculation of septic material is reduced to almost a minimum.

## Figures and Tables

**A B C D E F G H I f1:**